# Fine-scale spatial and temporal heterogeneities in insecticide resistance profiles of the malaria vector, *Anopheles arabiensis* in rural south-eastern Tanzania

**DOI:** 10.12688/wellcomeopenres.12617.1

**Published:** 2017-10-02

**Authors:** Nancy S. Matowo, Givemore Munhenga, Marcel Tanner, Maureen Coetzee, Wim F. Feringa, Halfan S. Ngowo, Lizette L. Koekemoer, Fredros O. Okumu

**Affiliations:** 1 Wits Research Institute for Malaria, MRC Collaborating Centre for Multi-disciplinary Research on Malaria, School of Pathology, Faculty of Health Sciences, University of the Witwatersrand, Johannesburg, 2000, South Africa; 2 Environmental Health and Ecological Sciences Department, Ifakara Health Institute, Ifakara, Tanzania; 3 Centre for Emerging Zoonotic and Parasitic Diseases, National Institute for Communicable Diseases, Johannesburg, 2131, South Africa; 4 University of Basel, Basel, 4001, Switzerland; 5 Swiss Tropical and Public Health Institute, Basel, 4051, Switzerland; 6 Faculty of Geo-Information Science and Earth Observation, University of Twente, Enschede, 7522 NB, Netherlands; 7 Institute of Biodiversity, Animal Health and Comparative Medicine, University of Glasgow, Glasgow, G12 8QQ, UK; 8 School of Public Health, Faculty of Health Sciences, University of the Witwatersrand, Johannesburg, 2000, South Africa

**Keywords:** Spatial and temporal variations, insecticide resistance, metabolic resistance, kdr detection, malaria control, Anopheles arabiensis, Tanzania

## Abstract

**Background: **Programmatic monitoring of insecticide resistance in disease vectors is mostly done on a large scale, often focusing on differences between districts, regions or countries. However, local heterogeneities in residual malaria transmission imply the need for finer-scale data. This study reports small-scale variations of insecticide susceptibility in *Anopheles arabiensis* between three neighbouring villages across two seasons in Tanzania, where insecticidal bed nets are extensively used, but malaria transmission persists.

**Methods: **WHO insecticide susceptibility assays were conducted on female and male *An. arabiensis* from three proximal villages, Minepa, Lupiro, and Mavimba, during dry (June-December 2015) and wet (January-May 2016) seasons. Adults emerging from wild-collected larvae were exposed to 0.05% lambda-cyhalothrin, 0.05% deltamethrin, 0.75% permethrin, 4% DDT, 4% dieldrin, 0.1% bendiocarb, 0.1% propoxur, 0.25% pirimiphos-methyl and 5% malathion. A hydrolysis probe assay was used to screen for L1014F ( *kdr-w*) and L1014S ( *kdr-e*) mutations in specimens resistant to DDT or pyrethroids. Synergist assays using piperonly butoxide (PBO) and triphenol phosphate (TPP) were done to assess pyrethroid and bendiocarb resistance phenotypes.

**Results: **There were clear seasonal and spatial fluctuations in phenotypic resistance status in *An. arabiensis* to pyrethroids, DDT and bendiocarb. Pre-exposure to PBO and TPP, resulted in lower knockdown rates and higher mortalities against pyrethroids and bendiocarb, compared to tests without the synergists. Neither L1014F nor L1014S mutations were detected.

**Conclusions: **This study confirmed the presence of pyrethroid resistance in *An. arabiensis* and showed small-scale differences in resistance levels between the villages, and between seasons. Substantial, though incomplete, reversal of pyrethroid and bendiocarb resistance following pre-exposure to PBO and TPP, and absence of *kdr *alleles suggest involvement of P450 monooxygenases and esterases in the resistant phenotypes. We recommend, for effective resistance management, further bioassays to quantify the strength of resistance, and both biochemical and molecular analysis to elucidate specific enzymes responsible in resistance.

## Introduction

In sub-Saharan Africa, malaria vector control relies predominantly on insecticide-based, methods, namely long-lasting insecticide treated bed nets (LLINs) and indoor residual spraying (IRS) of households. In Tanzania, LLINs are widely distributed and used as the primary and most affordable protective measure against diseases vectors ^1– 3^. The country has also recently implemented IRS, as a complementary vector control intervention in the north-western regions, with 11.6% – 14% of households currently covered by IRS ^4, 5^. Globally, implementation of LLINs and IRS, coupled with improved case diagnosis and treatment, as well as urbanization, improved living standards, and overall improvements in health systems, have contributed to 37% and 60% reduction of malaria morbidity and mortality respectively, between 2000 and 2015 ^6^. In Tanzania, high malaria transmission remains, with an average prevalence of 14.8% in children under 5 years ^7^. Nevertheless, the National Malaria Control Program currently has a strategic goal of reducing malaria prevalence to 1% by 2020 ^8^.

Despite the recent successes, efficacy of current malaria interventions is hampered by numerous challenges, particularly insecticide resistance in malaria vectors ^9– 11^. This has necessitated continuous insecticide resistance monitoring and periodic changes of insecticides used ^12– 15^. Some countries have put in place mechanisms to monitor susceptibility of malaria vectors to insecticides using guidelines provided by the World Health Organization (WHO) Global Plan for Insecticide Resistance Monitoring (GPIRM) ^16^. However, due to limited resources, insecticide resistance monitoring is mainly carried out only at large scale, often focusing on differences between districts or regions ^9, 14^. In Tanzania, insecticide susceptibility monitoring in mosquito populations is conducted at district level, relying on designated sentinel sites in regions, considered to be representative of the whole country ^14, 15^. Such a generalized approach to insecticide resistance monitoring is not very effective to capture local variations, where there might be pockets of high and low malaria transmission areas ^17, 18^. The variations may be due to, among other factors, impacts of interventions or genetic differences in mosquito populations, in turn resulting in physiological differences in response to insecticidal pressures ^17, 18^.

Different mosquito populations respond differently to insecticide pressure, depending on presence or absence, and type of resistance genes prevalent in the population ^19– 21^. This results in occurrence of geographically distinct populations, which might result in transmission variability over space and time. It is likely that these fine scale-variabilities are associated with the occurrence of residual mosquito biting “hotspots”, contributing to persistent residual malaria transmission in areas where LLINs and IRS are already widely used ^18^. Despite this, most vector surveillance programs still use global approaches without taking population variability into consideration. Furthermore, insecticide resistance studies have mainly focused on adult female mosquitoes, with limited studies on male populations.

The present study aimed at evaluating insecticide susceptibility of the dominant malaria vector, *An. arabiensis*, at a fine-scale between nearby villages in south-eastern Tanzania, where insecticides have been widely used for public health and agriculture, but where malaria transmission still persists.

## Methods

### Study villages

**Figure 1.**
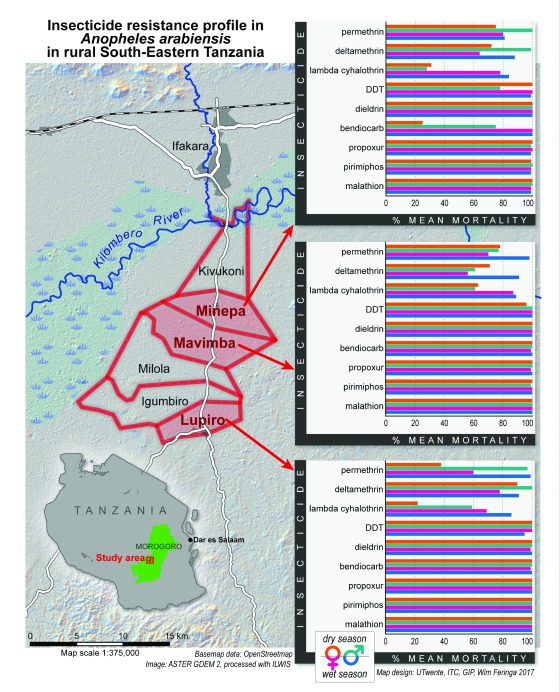
Geographic positions of the three study villages in south-eastern Tanzania. Embedded charts represent the fine-scale spatial and temporal variations of insecticide resistance profiles in both male and female malaria mosquitoes between the study villages.

Sampling of mosquito larvae was carried out in three proximal villages of Minepa (-8.2665°S, 36.6775°E), Lupiro (- 8.3857°S, 36.6791°E), and Mavimba (- 8.3163°S, 36.6810°E), located in Ulanga district, south-eastern Tanzania ( Figure 1). The minimum distance between villages was ~4km from Minepa to Mavimba, while the maximum distance was 9km from Minepa to Lupiro. All the villages lie between 120 and 350 meters above sea level, and are located in the flood plains of the Kilombero river, between the Udzungwa mountain ranges to the north, and Mahenge hills to the south ^1– 3^. The main economic activity of the area is irrigated rice farming. The irrigation leaves rice paddies continuously flooded, creating permanent water bodies favourable for mosquito breeding habitats. It is also a perennially meso-endemic malaria area, where transmission is predominantly by *An. funestus s.s and An. arabiensis* ^22– 25^. Recent multiple assessments conducted in the same area have revealed that 100% of the *An. gambiae s.l* mosquitoes in this study area were *An. arabiensis* sibling species ^25, 26^. As such, all field-collected *An. gambiae s.l* mosquitoes are henceforth referred to as *An. arabiensis.* The main malaria vector control intervention in the area is LLINs ^1– 3^.

### Mosquito sampling and rearing

Larval collections were carried out in the dry season between June and December 2015, and in the wet season between January and May 2016. For each village, between seven and nine breeding sites were identified, geo-referenced, and permanently established as larval sampling points for resistance monitoring during this study. Immediately after sampling, larvae were separated into anophelines and culicines to prevent cannibalism, and for easier adult morphological identifications. After morphological identification, larvae were pooled by village and reared into adults under standard insectary conditions (temperature of 27 ± 3°C and relative humidity 70–90%) in a semi-field screen house ^27^. During rearing, larvae were fed on mud, and algae collected from the respective breeding sites, and supplemented with Tetramin ^®^ fish food (Tetra, Melle, Germany). Each morning, pupae were transferred into a plastic cup and placed in a net-covered cage for adult emergence. After emergence, adults were separated by sex, transferred into individual small cages with provision of 10% glucose solution and maintained at 27–28°C and relative humidity of 70–90% for subsequent bioassays.

### Insecticide susceptibility tests

Phenotypic resistance tests on adults were conducted following WHO guidelines ^28^. Prior to susceptibility tests, efficacy of insecticide impregnated papers was verified against a known laboratory-reared susceptible *An. gambiae s.s.* strain (Ifakara strain) ^29, 30^. A group of 20 – 25 non-blood fed wild female and male mosquitoes aged three to five days were exposed for an hour to the diagnostic concentrations of 0.05% lambda-cyhalothrin, 0.05% deltamethrin, 0.75% permethrin, 4% DDT, 4% dieldrin, 0.1% bendiocarb, 0.1% propoxur, 0.25% pirimiphos-methyl, and 5% malathion. Controls consisted of mosquitoes exposed to oil-impregnated papers. During the one hour exposure to insecticides, knockdown rates were recorded at 10, 15, 20, 30, 40, 50, and 60 minute intervals. After the exposure period, mosquitoes were transferred to holding tubes and maintained on 10% glucose solution. The final mortalities were recorded 24 hours post-exposure. Dead and surviving mosquitoes were kept separately, under preservation using silica in 1.5 ml Eppendorf tubes, for further molecular examination of resistance genes.

### Synergist bioassays

Synergist bioassays using piperonyl butoxide (PBO), an inhibitor of monooxygenase, and triphenyl phosphate (TPP), an inhibitor of esterases, were performed on the adult mosquitoes, to assess whether the pyrethroid resistance phenotypes observed during WHO susceptibility assays could be reversed by synergistic activity of these insecticides, which would indicate a biochemical basis for the resistance ^28, 31^. Prior to the synergist assays, the bio-efficacy and quality of PBO and TPP synergist papers was validated against a reference laboratory colony, whose pyrethroid resistance and DDT resistance is mediated by high monooxygenases (FUMOZ-R) ^32^ and elevation of esterases (MBN-DDT) ^33^, respectively.

Due to limited number of mosquito sample, the PBO and TPP assays were performed only on female *An. arabiensis* collected from Minepa village, and PBO test only in female *An. arabiensis* sampled from Mavimba village between the months of September and December 2016. Non-blood fed, 2–3 day old wild female *An. arabiensis* mosquitoes were used, each test consisting of 20 to 25 mosquitoes per tube with two controls. Five replicates were performed for each exposure set. Mosquitoes were pre-exposed to (either 4% PBO or 20% TPP) for 60 minutes, followed by exposure to WHO test papers impregnated with discriminatory doses of candidate insecticides (0.75% permethrin, 0.05% deltamethrin, 0.05% lambda-cyhalothrin, or 4% DDT) for another 60 minutes. To assess the effect of insecticides alone, another group of mosquitoes without pre-exposure to the synergists were concurrently exposed to each candidate insecticide only. At the same time, the same number of mosquitoes was exposed to either 4% PBO or 20% TPP only. Another group of mosquitoes was also exposed to control filter papers treated with a mixture of olive oil and acetone, and to plain filter papers with no chemicals that were used as environmental controls. During the one hour exposure to synergist and to insecticides, the knock-down rates were recorded at 5,10,15,20,25,30,40,50 and 60 minute intervals. Mosquitoes were fed on 10% glucose solution, and mortalities from assays conducted with and without exposure to synergist were scored 24 hours post-exposure ^31^.

### Knockdown resistance (kdr) detection using hydrolysis probe analysis

A hydrolysis probe assay was used to screen for L1014F ( *kdr-w*) and L1014S ( *kdr-e*) mutations in 220 randomly selected dead and alive female specimens, which had shown resistance to both DDT or pyrethroids, using procedures previously described ^33^. DNA was extracted from the legs of each specimen using the ZyGEm prepGEM insect DNA extraction kit (Cat: PIN141106, ZyGEM NZ Ltd, Ruakura, New Zealand), following the manufacturer’s guidelines, except that the reaction volume was quartered. DNA extracted (10–50ng) from each individual mosquito was then used to detect the presence of *kdr-w* and *kdr-e* in two PCR master mixtures in a CFX 96 real-time PCR machine (Biorad, Hercules, CA, USA). In each instance, positive controls comprised of a DNA template from mosquitoes with known West African ( *kdr-w*) genotype sampled from Sudan (SENN-DDT, homozygous for the L1014F mutation) ^34^, and DNA from Burundi mosquitoes, which had been previously genotyped as homozygous for the East African ( *kdr-e*) mutation, L1014S (unpublished study, Vector Control Reference Laboratory, Johannesburg, South Africa). Other positive controls were DNA templates from a homozygous susceptible colony originating from Kanyemba, Zimbabwe (KGB). The heterozygous controls were made up by mixing equal aliquots of susceptible and resistant DNA templates. A final control consisted of a master mix containing of PCR components, except the DNA template that was set up to monitor any contamination during reaction preparation.

### Data analysis

Data analysis was done using R version 3.0 ^35^. Susceptibility bioassay data was first summarised as mean percentage (%) mortality per insecticide per village and per season. Population susceptibility was classified according to the WHO criteria ^28^. Data for the synergist tests were summarized as mean % mortality of the four replicates, and the 95% confidence intervals were calculated to estimate probability that population means lie within the given ranges. Following an average of four replicates of each synergist test, final mortality observed 24 hours post-exposure was compared between samples with and without pre-exposure to synergists, using paired sample t-test. The time at which 50% of the experimental populations were knocked down (KDT _50_) was determined using log-probit analysis ^36^. Resistance reduction was obtained by dividing the KDT _50_ obtained from insecticide exposure with no synergist by the KDT _50_ obtained from insecticide plus the synergist (KDT _50 Insecticide alone_/KDT _50 Insecticide plus Synergist_). The differences in mortality was considered statistically significant when P< 0.05. For *kdr* detection assays, the fluorescent signals detected in the experimental reactions were compared to those of the controls, and genotyping of each mosquito was done using the CFX manager software version 2.1 (Bio-Rad, Hercules, CA, USA).

### Ethical statement

Permission to conduct larva sampling was obtained from the owners of the farms, after the researchers provided a description of the study aims and procedures. A brief description of the study was delivered in local language, Kiswahili. Upon agreement, participants were asked to sign written informed forms. The proposed study went through an ethical review and obtained approval from the institutional review board of Ifakara Health Institute (Ref: IHI/IRB/NO: 34-2014) and the Medical Research Coordinating Committee at the National Institute for Medical Research in Tanzania (Ref: NIMR/HQ/R.8a/Vol.IX/1903). Permission to publish this manuscript was obtained from the National Institute for Medical Research in Tanzania (NIMR; Ref: NIMR/HQ/P.12 VOL. XXII/27). Printed copies and online links to the manuscript will be provided to NIMR upon publication.

## Results

### Spatial and seasonal variability in phenotypic resistance in male and female *An. arabiensis* mosquitoes

The reference insectary-reared *An. gambiae ss* were fully susceptible (100% mortality) to all the insecticides tested, confirming the quality and bio-efficacy of the insecticide-impregnated papers used. The observed mortality in control groups was consistently below 5%, so no statistical correction was required. The WHO susceptibility test findings are summarized in Figure 1 and Table 1. There was marked seasonal and spatial variations in phenotypic resistance in both female and male *An. arabiensis* to three pyrethroids, permethrin, deltamethrin, lambda-cyhalothrin, but also to bendiocarb and DDT in the study villages.

**Table 1.** Mean percentage mortalities following exposure to insecticides for samples of 2–5 day old *Anopheles arabiensis* adults emerging from larval collections done in the dry season (June–December 2015) and the wet season (January–May 2016) from three neighbouring villages. Mortalities were recorded 24 hours post exposure. Tests showing seasonal or spatial variability in susceptibility status are marked with symbols, ++ or ^^.

		Minepa Village % mean mortality (95% CI)		Mavimba Village % mean mortality (95% CI)		Lupiro Village % mean mortality (95% CI)	
	Insecticides	Dry Season (n=1298)	Wet Season (n= 1200)	Dry Season (n=1345)	Wet Season (n= 1082)	Dry Season (n= 1318)	Wet Season (n=1115)
Female Mosquitoes	0.75% Permethrin	75.4 (64.1-84.0) ^RR^	80.6 (69.4-88.4) ^RR^	79.2 (65.4-88.5) ^RR^	70.1 (58.7-79.4) ^RR^	37.7 (26.8-50.1) ^RR^	60.0 (48.9-70.1) ^RR^
	0.05% Deltamethrin	72.4 (60.8-81.6) ^RR^	64.4 (51.2-75.6) ^RR^	72.1 (56.8-83.5) ^RR^	56.3 (44.7-67.2) ^RR^	90.3 (80.8-95.4) ^RS^	77.5 (67.1-85.3) ^RR^
	0.05% Lambda- cyhalothrin	31.1 (21.2-43.0) ^RR^	63.3 (50.2-74.8) ^RR^	63.2 (47.0-76.8) ^RR^	87.4 (78.3-89.8) ^RR^	21.6 (13.5-32.5) ^RR^	69.0 (57.8-77.9) ^RR^
	4% Dieldrin	100 ^SS^	100 ^SS^	100 ^SS^	100 ^SS^	100 ^SS^	98.8 (91.7-99.8) ^SS^
	4% DDT ++ ^^	100 ^SS^	100 ^SS^	96.5 (89.7-98.8) ^RS^	98.8 (91.6-99.8) ^SS^	100 ^SS^	83.5 (72.6-89.4) ^RR^
	0.1% Propoxur	100 ^SS^	100 ^SS^	100 ^SS^	98.8 (91.6-99.8) ^SS^	100 ^SS^	100 ^SS^
	0.1% Bendiocarb ++ ^^	24.6 (16.0-35.9) ^RR^	100 ^SS^	100 ^SS^	100 ^SS^	100 ^SS^	100 ^SS^
	0.25% Pirimiphos methyl	99.0 (93.0-99.9) ^SS^	99.1 (93.4-99.9) ^SS^	100 ^SS^	100 ^SS^	100 ^SS^	100 ^SS^
	5% Malathion	100 ^SS^	100 ^SS^	100 ^SS^	100 ^SS^	100 ^SS^	100 ^SS^
	Control (untreated paper)	1.6 (0.7-3.4)	3.1 (1.3-7.0)	0.6 (0.1-2.7)	1.3 (0.5-3.2)	3.9 (2.2-7.2)	1.9 (0.9 – 3.9)
		**(n= 1326)**	**(n=1198)**	**(n=1276)**	**(n=1080)**	**(n=1298)**	**(n=1110)**
Male Mosquitoes	0.75% Permethrin ++ ^^	100 ^SS^	80.5 (69.4-88.3) ^RR^	77.4 (62.4-87.6) ^RR^	97.5 (90.6-99.4) ^SS^	97.2 (88.8-99.3) ^RS^	98.8 (91.8-99.8) ^SS^
	0.05% Deltamethrin ^^	98.5 (92.7-99.7) ^SS^	87.5 (78.0-93.2) ^RR^	60.8 (43.8-75.5) ^RR^	91.3 (82.8-95.8) ^RS^	100 ^SS^	90.6 (81.6-95.4) ^RS^
	0.05% Lambda- cyhalothrin ++ ^^	28.1 (14.2-48.0) ^RR^	83.5 (73.0-90.5) ^RR^	60.6 ^RR^	89.4 (18.8-38.3) ^RR^	58.9 (32.4-81.1) ^RR^	85.7 (75.1-92.3) ^RR^
	4% Dieldrin	100 ^SS^	100 ^SS^	100 ^SS^	100 ^SS^	100 ^SS^	100 ^SS^
	4% DDT ++ ^^	78.4 (60.2-89.7) ^RR^	99.1 (93.4-99.9) ^SS^	100 ^SS^	100 ^SS^	100 ^SS^	95.3 (87.2-98.4) ^RS^
	0.1% Propoxur	99.2 (93.9-99.9) ^SS^	99.1 (93.4-99.9) ^SS^	100 ^SS^	100 ^SS^	100 ^SS^	100 ^SS^
	0.1% Bendiocarb ++ ^^	75.3 (56.2-87.9) ^RR^	100 ^SS^	100 ^SS^	100 ^SS^	99.3 (93.7-99.9) ^SS^	100 ^SS^
	0.25 % Pirimiphos methyl	100 ^SS^	99.1 (93.4-99.9) ^SS^	99.1 (93.6-99.9) ^SS^	100 ^SS^	100 ^SS^	100 ^SS^
	5% Malathion	100 ^SS^	99.1 (93.4-99.9) ^SS^	100 ^SS^	100 ^SS^	100 ^SS^	100 ^SS^
	Control (untreated paper)	1.4 (0.5-4.0)	1.4 (0.5-4.2)	1.2 (0.5-3.2)	2.8 (1.5-5.1)	3.8 (1.1-12.4)	0.9 (0.3-2.7)

SS: Mosquitoes were susceptible to the test insecticide (WHO assays mortality between 98% and 100%). RS: Mosquitoes had reduced susceptibility indicating possible resistance and need for further investigation (WHO assays mortality of 90% to 97%). RR: Mosquitoes were confirmed resistant to the test insecticide (WHO assays mortality below 90%). ++ Insecticides for which we observed differences in susceptibility of *Anopheles arabiensis* mosquitoes between dry and wet seasons, i.e. where mosquitoes were fully susceptible in one season and fully resistant in a different season in same village. ^^ Insecticides for which we observed differences in susceptibility of *Anopheles arabiensis* mosquitoes between (nearby) villages, i.e. where mosquitoes were fully susceptible in one village and fully resistant in another.

For example, in Minepa village, the female mosquitoes were fully susceptible to bendiocarb in the wet season (mean mortality of 100%), yet highly resistant in the dry season (24.6%). Bendiocarb resistance also varied across different locations. While females collected from Minepa village in the dry season were resistant to bendiocarb, samples of the same species collected from the nearby villages of Mavimba and Lupiro during the same season were fully susceptible to the same chemical (100%). It was also observed that female *An. arabiensis* mosquitoes collected from Minepa village were fully susceptible to DDT (100%) in both seasons, while those collected from the nearby Mavimba village during dry season showed reduced susceptibility to DDT (96.5%), and resistance to the same insecticide in Lupiro village in the wet season (83.5%). Wild female mosquito populations from Minepa, Mavimba and Lupiro villages displayed variable levels of deltamethrin resistance across both seasons, but reduced susceptibility to this insecticide (90.3%) in dry season in Lupiro. Throughout the study, female *An. arabiensis* were resistant to permethrin and lambda-cyhalothrin (mortality rates between 21.6% and 87.4%) in both seasons across the study villages.

As shown in Table 1, insecticide resistance variation in the male *An. arabiensis* was greater both by season and by locality, and was observed for pyrethroids, DDT and bendiocarb. Males collected from Minepa were fully susceptible to permethrin in the dry season (100% mortality), but resistant to the same chemical in the wet season (80.5% mortality); those collected from Mavimba village on the other hand were susceptible to permethrin in the wet season (97.5%), but resistant in dry season (77.4%). In Lupiro village, the males were fully susceptible to permethrin in wet seasons (98.8%), though there were also signs of weakening susceptibility among mosquitoes collected in dry season (97.2%). Deltamethrin resistance in male *An. arabiensis* was observed in wet season in Minepa (87.5%) and in dry season in Mavimba (60.8%). There was also reduced susceptibility to deltamethrin in the male mosquito population sampled from Mavimba (91.3%) and Lupiro (90.6%) in wet season, but complete susceptibility was observed in Minepa (98.5%) and Lupiro in dry season (100%).

During the study, male mosquito samples from the three villages across both seasons displayed various levels of resistance to lambda-cyhalothrin (mortality rates between 28.1% and 89.4%). For both DDT and bendiocarb, male mosquitoes from Minepa were resistant in dry season 78.4% and 75.3% respectively, but susceptible in wet season (mortalities between 99.1% and 100%), while the males from both Mavimba and Lupiro were consistently susceptible to these two insecticides in both seasons (100%). A minor exception was specimens collected in wet season from Lupiro, where reduced susceptibility was observed against DDT (95.3%).

As illustrated in Figure 1, both male and female mosquito populations across the study villages and during both seasons remained fully susceptible to propoxur, dieldrin and all organophosphates tested (mortality rates between 98.8% and 100%).

### Results of the synergist bioassays conducted with samples from Minepa village

***Tests with PBO.*** There was a reduction in time to 50% knockdown (KDT _50_) in mosquito cohorts pre-exposed to PBO followed by deltamethrin, permethrin, lambda cyhalothrin and bendiocarb), compared to cohorts directly exposed to each of the candidate insecticides without PBO pre-exposure ( Table 2). Resistance reduction levels of 1.4, 3.1, 1.9 and 1.5 fold were recorded in tests of deltamethrin, permethrin, lambda-cyhalothrin and bendiocarb, respectively. The resistance reduction ratios for all tested insecticides are shown in Table 2.

**Table 2.** Knockdown rates (KDT _50_) and degree of resistance reduction of *Anopheles arabiensis* from two study villages after being exposed to various insecticides with and without pre-exposure to synergists.

Study sites	Insecticide	KDT _50_ (min)	(95% CI)	Resistance reduction ^¥^
Minepa village	0.05% Deltamethrin	50.24	35.71 – 64.77	-
	4% PBO + 0.05% Deltamethrin	35.90	27.56 – 44.24	1.40
	0.75% Permethrin	70.20	34.42 – 105.98	-
	4% PBO +0.75% Permethrin	22.72	17.82 – 27.61	3.09
	0.05% Lambda cyhalothrin	54.88	34.62– 75.13	-
	4% PBO + 0.05% Lambda cyhalothrin	29.61	22.55 - 36.66	1.85
	0.05% Deltamethrin	60.87	38.84 – 82.89	-
	20% TPP + 0.05% Deltamethrin	65.23	35.46 – 94.99	0.93
	0.75% Permethrin	38.69	30.60 – 46.77	-
	20% TPP +0.75% Permethrin	27.65	20.59 – 34.70	1.40
	0.1% Bendiocarb	53.14	37.00 – 69.28	-
	4% PBO + 0.1% Bendiocarb	35.25	27.51 – 42.99	1.51
	0.1% Bendiocarb	56.14	40.04 – 72.24	-
	20% TPP +0.1% Bendiocarb	43.71	33.44 – 53.98	1.28
Mavimba village	0.05% Deltamethrin	46.35	32.64 – 60.06	-
	4% PBO + 0.05% Deltamethrin	23.33	17.98 – 28.68	1.99
	0.75% Permethrin	39.78	29.16 – 50.39	-
	4% PBO + 0.75% Permethrin	21.09	15.60 – 26.59	1.89
	0.05% Lambda cyhalothrin	68.65	35.10 – 102.20	-
	4% PBO +0.05% Lambda cyhalothrin	34.87	26.82 – 42.93	1.97

^¥^ Resistance reduction = KDT _50 insecticide alone_/KDT _50 insecticide plus synergist_

There was also a significant difference in 24-hr post-exposure mortality between mosquito cohorts ( Table 3). Our tests revealed significant increases in mortalities when the mosquito populations were pre-exposed to PBO followed by deltamethrin compared to when the same populations were exposed to deltamethrin alone (paired t-test, df = 3, t = 18.4, and P < 0.001). Pre-exposure to PBO followed by permethrin also resulted in a significant increase in mortality relative to exposure to permethrin alone (paired t-test, df = 3, t = 9.80, and P = 0.002). Similarly, pre-exposure to PBO followed by lambda cyhalothrin yield a significant increase in mortality compared to cohorts exposed to lambda cyhalothrin alone (paired t-test, df = 3, t = 10.3, and P = 0.002). In tests for bendiocarb resistance, it was observed that pre-exposure to PBO created substantial synergism, resulting in higher mortality compared to exposure to bendiocarb with no synergist (paired t-test, df =3, t = 22.46, and P < 0.001).

**Table 3.** Mortality *Anopheles arabiensis* from Minepa village exposed to insecticides and the synergists, PBO or TPP.

Treatment	No. replicates done	Sample size *		% mean mortality (95% CI)		
			Minepa village			
			0.05% Deltamethrin	0.05% Lambda cyhalothrin	0.75% Permethrin	0.1% Bendiocarb
Environmental control	4	375	0	NA	0	0
Solvent control	4	375	0	NA	0	0
20% TPP only	4	375	0	NA	0	0
20% TPP & Test insecticide	4	375	27.0 (18.3 – 35.7) ^b^	NA	29.5 (20.3 – 38.7) ^b^	72.0 (62.9 – 81.0) ^a^
Test insecticide only	4	374	24.0 (13.4 – 34.6) ^b^	NA	26.5 (21.1 – 31.9) ^b^	55.5 (46.4 – 64.6) ^a^
Environmental control	4	370	0.2 (-0.2 – 0.6)	0	0	0
Solvent control	4	370	0.2 (-0.2 – 0.6)	0	0	0
4% PBO only	4	370	0	0	0	0
4% PBO & Test Insecticide	4	370	73.0 (63.5 – 82.5) ^b^	97.5 (94.7 – 100) ^a^	56.8 (46.9 – 66.6) ^a^	76.0 (60.4 – 91.6) ^a^
Test Insecticide only	4	370	45.0 (35.5 – 54.5) ^b^	20.0 (5.6 – 34.4) ^a^	08.8 (03.0 – 14.1) ^a^	33.0 (23.5 – 42.5) ^a^

NA=No assay was performed on this insecticide. ^a ^There are significant differences in mean mortalities between exposure to insecticides with and without synergists. ^b^No significant difference in mean mortalities between exposure to insecticides with and without synergists.

***Tests with TPP.*** There was a slight decrease in KDT _50_ when mosquitos were pre-exposed to TPP followed by either deltamethrin, permethrin or bendiocarb, compared to when the same population of mosquitoes was exposed to the candidate insecticides alone ( Table 2). Resistance to deltamethrin, permethrin, and bendiocarb were reduced by 0.9, 1.4, and 1.3 fold, respectively, with TPP ( Table 2). However, there was no difference in mortalities in mosquitoes exposed to deltamethrin with or without pre-exposure to TPP (paired t-test, df = 3, t = 0.73, and P = 0.520). Also, there was no statistical difference in mean mortalities of mosquitoes exposed to TPP plus permethrin compared to when they were exposed to permethrin alone (paired t-test, df = 3, t = 0.88, and P = 0.444). On the other hand, there were differences in the mean mortality between bendiocarb and TPP + bendiocarb (paired t-test, df = 3, t = 19.12, and P = 0.006).

### Results of the synergist bioassays conducted with samples from Mavimba village

Prior exposure to PBO partially restored susceptibility to deltamethrin by 2.0 fold and decreased the KDT _50_ from 46.35min for deltamethrin alone to 23.33 min for deltamethrin and PBO ( Table 2). The time required for 50% of the mosquitoes to be knocked down was also reduced from 39.78min for permethrin alone to 21.09 min after being exposed for permethrin and PBO. Resistance reduction level for permethrin following PBO pre-exposure was 1.9 fold ( Table 2). Similarly, the resistance to lambda-cyhalothrin was reduced by 2.0 fold with PBO, with a shift in KDT _50_ from 68.65min to 34.87min ( Table 2). There was a significant increase in mortality in mosquito populations pre-exposed to PBO followed by deltamethrin compared to when the same populations were exposed to deltamethrin alone (paired t-test, t = 18.4, df =3, p < 0.001) ( Table 4). Similarly, when the mosquito populations were pre-exposed to PBO followed by lambda-cyhalothrin this resulted in a significant increase in mean mortality compared to when the same population was exposed to lambda cyhalothrin alone (paired t-test, t = 17.9, df = 3, p < 0.001) ( Table 4).

**Table 4.** Mortality *Anopheles arabiensis* from Mavimba village exposed to insecticides and the synergists, PBO.

Treatment	No. replicates done	Sample size*	% mean mortality (95% CI)		
			Mavimba village		
			0.05% Deltamethrin	0.05% Lambda cyhalothrin	0.75% Permethrin
Environmental control	4	260	0.4 (-0.4 – 1.2)	0.4 (-0.4 – 1.2)	0
Solvent control	4	262	0.3 (-0.3 – 0.9)	0	0
4% PBO only	4	262	0	0	0
4% PBO & Test Insecticide	4	241	92.5 (86.2 – 98.8) ^a^	85.2 (74.6 – 95.8) ^a^	91.3 (82.9 – 99.6) ^a^
Test Insecticide only	4	240	27.5 (24.7 – 30.3) ^a^	20.0 (03.5 – 36.5) ^a^	67.5 (54.5 – 80.5) ^a^

^a^ There are significant differences in mean mortalities between exposure to insecticides with and without synergists. ^b^ No significant difference in mean mortalities between exposure to insecticides with and without synergists

### Results of the molecular assays to detect knockdown resistance (kdr) alleles

A total of 74 adult female *An. arabiensis* mosquitoes from Minepa, 66 from Mavimba and 80 from Lupiro were assayed for *kdr* allele mutations L1014F ( *kdr-west*) and the L1014S ( *kdr-east*). All specimens were negative for both mutations.

## Discussion

The increasing spread of insecticide resistance in malaria vectors jeopardizes control and elimination efforts ^9– 14^, thus necessitating regular resistance monitoring to design setting-specific and successful resistance management programmes ^16, 28, 37^. Overall, this study detected widespread resistance against pyrethroids, bendiocarb, and DDT; but not against propoxur, dieldrin, and the two organophosphates, pirimiphos-methyl and malathion, for which there was full susceptibility across all the villages and seasons. This study also found marked temporal and fine-scale fluctuations of insecticide resistance profiles in both male and female *An. arabiensis* against three insecticides in the pyrethroid class, DDT, and bendiocarb. In all the three villages, deltamethrin, permethrin, lambda-cyhalothrin, DDT and bendiocarb resistance of male *An. arabiensis* mosquitoes fluctuated between seasons and villages. Resistance of female *An. arabiensis* mosquitoes against DDT and bendiocarb also fluctuated between seasons and villages. The most resistant populations were observed in Minepa for bendiocarb, lambda-cyhalothrin and DDT and in Lupiro for lambda-cyhalothrin and permethrin. In Minepa, bendiocarb resistance was detected in the dry season, but completely diminished in wet seasons for both male and female populations, and DDT resistance followed a similar trend in the male population. However, in Lupiro village, DDT resistance was observed during the wet season only.

The seasonal and spatial variation in insecticide resistance detected in this study is not unique. Variations in both phenotypic and genotypic insecticide resistance in both *Anopheles* and *Aedes* mosquitoes over small spaces and time have been reported previously ^22, 38– 40^. A recent report in Chad found a significant spatial changes in insecticide resistance in an *An. arabiensis* population ^38^. Similarly, there was significant difference in phenotypic and genotypic resistance at a fine geographical scale in *Ae. aegypti* populations to chlorpyrifos-ethyl and deltamethrin sampled from nearby study sites in Mexico ^39^. The seasonal and spatial fluctuations in insecticide resistance might be attributed to differences in the biology and genetics of the vector populations in particular ecological settings, as reported in a previous study by Verhaeghen *et al.* ^40^.

Perhaps the presence of chemical contaminants in a particular environment, possibly due to leached agricultural chemicals and other pollutants at a particular time might cause selection pressure in mosquitoes, and subsequent resistance to insecticides. Also, the existence of phenotypic resistance in the study areas to lambda-cyhalothrin, bendiocarb and DDT that are not used for LLINs or IRS, suggest cross-resistance between classes or alternative sources of insecticide resistance pressure, most likely from agriculture. The impact of agricultural pesticides in the selection of resistant mosquitoes has already been reported extensively ^19, 41– 48^. This hypothesis is also supported by our preliminary observations that the majority of farmers in the study villages reported applying more pesticides in dry seasons than in wet seasons (Matowo N, Munhenga G, Tanner M, Koekemoer L, Coetzee M and Okumu F *,* unpublished study, Ifakara Health Institute). The differences in insecticide resistance between adjacent study villages suggests that other than variations that have been reported between districts and regions ^10, 14, 15^, there might also be fine-scale differences even within the villages that require further investigations. All these variations signify an important challenge to the vector control programs that might require proper consideration in the timing/season and choosing different insecticides for application even in a particular small area.

Male mosquitoes are considered to be more delicate and susceptible to insecticides as they have a shorter life expectancy than their females counterparts ^28^. In this study, males were found to be resistant to the same insecticides as the females, but at a lower level. These observations are consistent with previous studies that have reported that adults male *An. arabiensis*, with previous exposure to insecticides, could also experience resistance similar to females ^49^. For example, a high level of glutathione-S-transferase (GSTs) activity was found in both male and female *An. arabiensis* selected for resistance to DDT, but only elevated esterases was found in the male-DDT selected strain ^49^. Resistance in male mosquitoes was reported previously to adversely affect their mating competiveness, as shown in *Culex pipiens* and *An. gambiae* ^50– 52^. This suggests the need for regular monitoring of susceptibility status of male mosquitoes, particularly in interventions targeting male mating behaviour, such as the sterile insect technique, which involves mass-rearing, sterilization, and release of sterile male mosquitoes into the wild population to prevent females from reproducing ^53, 54^. Other interventions that have been proposed for mosquito-borne disease elimination includes targeting male swarming behaviour ^55^, sugar-seeking behaviour through the use of attractive toxic sugar baits ^56, 57^ and larval control ^58^. In summary, our findings and the current evidence suggest the need for regular monitoring of susceptibility status of both males and females, especially for end-game scenarios where LLINs and IRS have already been widely used, but malaria transmission still persists.

As revealed in the synergist assays, the reduction in knockdown rates and increase in mortalities was due to synergistic action of piperonal butoxide (PBO), as an inhibitor of P450 monooxygenases, and triphenol phosphate (TPP), as an inhibitor of the esterases activity. Synergists have an effect by augmenting the penetration of the insecticides into the mosquito body and counteracting the metabolic pathways that would otherwise metabolize the insecticides, thus restoring susceptibility to varying degrees ^31, 59– 61^. The observed effects in the present study suggest involvement to a significant degree of one or both of the two enzyme classes in conferring pyrethroid and bendiocarb resistance within the mosquito populations sampled from the study sites. However, esterases seem not to be involved in deltamethrin and permethrin resistance in the mosquito population sampled from Minepa village. Susceptibility to lambda-cyhalothrin was completely restored by 4% PBO in the mosquito population sampled from Minepa village, indicating that the resistance is metabolic mediated by monooxygenases. However, the inability of PBO and TPP to completely reverse the deltamethrin, permethrin and bendiocarb resistance across the study sites indicates that either other enzymes might be playing a role in the metabolic resistance, or there is presence of other mutations that require further investigation. These questions will need to be further explored through biochemical and genetic analyses. Our findings agree with previous studies that have consistently reported the combining effect of synergists and insecticides against resistant disease-transmitting mosquitoes and incomplete suppressions of pyrethroids resistance due to the synergists action ^17, 31, 59, 62– 64^.

The absence of L1014F and L1014S resistance alleles in the field-collected adult female mosquito populations suggests that the phenotypic resistance to pyrethroid and DDT was not associated with target site insensitivity of the voltage-gate sodium channel. The findings supports an earlier study by Okumu *et al.*, who also showed absence of *kdr* mutations in wild population of *An. arabiensis* from Lupiro village, five years before this current study ^65^. Similarly, a recent multi-region study in Tanzania by Kabula *et al.* ^66^ reported absence of both L1014F and L1014S mutations in *An. arabiensis* populations from Kilombero district, which neighbours Ulanga district where our study was conducted. However, these gene mutations were detected in both *An. arabiensis* and *An. gambiae s.s.* from other sentinel districts of Tanzania where studies were carried out ^66^.

## Conclusions

This study revealed multiple spatial and temporal fluctuations of insecticide resistance profiles in the *An. arabiensis* populations from the three neighbouring villages in south-eastern Tanzania, and confirmed the presence of pyrethroid, DDT and bendiocarb resistance in each of these three villages. The substantial, though not absolute reversal of pyrethroid and carbamate resistance when mosquitoes were pre-exposed to PBO or TPP, coupled with the absence of *kdr* resistance alleles, suggests involvement of P450 monooxygenases and esterases as key determinants conferring the resistance phenotypes. We recommend further intensity bioassays to determine the strength of phenotypic resistance, as well as biochemical and molecular analysis to elucidate various enzymes involved in the resistance. Such additional tests are essential for an effective resistance management programmes in this or similar areas. Overall, these results highlight the importance of periodic and continuous insecticide susceptibility surveillance and emphasize the need to consider fine-scale variations in insecticide resistance levels, even in small geographical locations, when implementing insecticidal-based interventions.

## Data availability

Raw datasets for this study are available from the Ifakara Health Institute data repository ( http://dx.doi.org/10.17890/ihi.2017.09.99 ^67^).
