# Supplementary material for: Fine-scale spatial and temporal heterogeneities in insecticide resistance profiles of the malaria vector, Anopheles arabiensis in rural south-eastern Tanzania
Source: Wellcome Open Res. 2017 Oct 2;2:96. [Version 1] doi: 10.12688/wellcomeopenres.12617.1 (PMC5782413; doi:10.12688/wellcomeopenres.12617.1)
Supplement: Peer review history

# Peer review: Referee response for version 1

**DOI:** 10.21956/wellcomeopenres.13662.r28984

**Published:** 2018-01-22

**Authors:** Delenasaw Yewhalaw

**Affiliations:** 1 Tropical and Infectious Diseases Research Center, Jimma University, Jimma, Ethiopia

**Comments to Authors**

 Title “Fine-scale spatial and temporal heterogeneities in insecticide resistance profiles of the malaria vector, *Anopheles arabiensis* in rural south-eastern Tanzania”

**General**

 This paper evaluated spatial and seasonal variations of insecticide resistance profile in the major malaria vector, *An. arabiensis*. Generally, this is a well written paper with important information showing how resistance level in malaria vectors vary periodically and among proximal localities in the same geographic area. The work further highlights the need to consider this small-scale variation in implementing insecticide resistance monitoring and management strategies which mainly relies on insecticide resistance data often coming from few selected district sentinel sites.  Such an approach is important for countries which initiated malaria elimination programs where residual transmission could be sustained due to resistance by local mosquito populations.

**Introduction**:

 OK, however, it would have been good to provide information on the type of insecticides which have been used for crop protection in the 3 villages as this may help understand the observed variation in resistance profile

**Methodology**:

 The methodology is sound and clear.  However, it is not clear why authors used 3-5 days old adult female mosquitoes for insecticide susceptibility test and 2-3 days old adult female mosquitoes for synergist assays.

**Results**:

 This reviewer would suggest to use “KD rate reduction” instead of “Resistance reduction”.  Authors should also interpret the results of PBO and TPP assays with caution as the results from these assays indicate partial involvement of P450 and esterases. Hence, those enzymes may not be key determinants conferring resistance in the mosquito populations. 

**Discussion**:

 OK

**Conclusion**

 OK

I have read this submission. I believe that I have an appropriate level of expertise to confirm that it is of an acceptable scientific standard.

# Peer review: Referee response for version 1

**DOI:** 10.21956/wellcomeopenres.13662.r26599

**Published:** 2017-10-05

**Authors:** Eric Ochomo

**Affiliations:** 1 Centre for Global Health Research, Kenya Medical Research Institute (KEMRI), Kisumu, Kenya

This manuscript is very well written and adequate in form and content. It could be improved greatly if the authors did the following:

 1. Methods: It is not clear whether the papers used for the WHO tube bioassays were procured from WHO sources or if these were made locally. if they were made locally, it would be nice to include a statement about QA and QC. 

 2. Results: The barcharts used to present resistance data should have error bars. 

 3. Table 2: The authors need to include some statistics of significance for the resistance reduction indices. 

 4. Discussion: One paper that would be useful to Cite is the Ochomo *et al *2014 paper which describes heterogeneity of insecticide resistance in Kenya ^1^.

I have read this submission. I believe that I have an appropriate level of expertise to confirm that it is of an acceptable scientific standard.

## References

[1] OchomoEBayohNMKamauLAtieliFVululeJOumaCOmbokMNjagiKSotiDMathengeEMuthamiLKinyariTSubramaniamKKleinschmidtIDonnellyMJMbogoC: Pyrethroid susceptibility of malaria vectors in four Districts of western Kenya. *Parasit Vectors*.2014;7: 10.1186/1756-3305-7-310 310 10.1186/1756-3305-7-310 24996418PMC4094666
